# Chronic myeloid leukemia in blast phase masquerading as T-lymphoblastic lymphoma with t(22;22) karyotype: a case report

**DOI:** 10.3389/fonc.2026.1784891

**Published:** 2026-04-13

**Authors:** Yang Liu, Zhengdong Hao, Jun Bai, Wen Zhou, Hongbin Zhang, Zhiyuan Guan, Liansheng Zhang, Lijuan Li

**Affiliations:** 1 Department of Hematology, Lanzhou University Second Hospital, Lanzhou University, Lanzhou, China; 2 Key Laboratory of the Hematology of Gansu Province, Lanzhou University Second Hospital, Lanzhou University, Lanzhou, China

**Keywords:** BCR::ABL1 fusion gene, blast phase chronic myeloid leukemia, complex karyotype, mixed phenotype, T-lymphoblastic lymphoma, tyrosine kinase inhibitor

## Abstract

This article reports a rare case of chronic myeloid leukemia (CML) in blast phase presenting clinically as T-lymphoblastic lymphoma (T-LBL). A 56-year-old male was admitted due to cervical masses, and lymph node biopsy initially diagnosed T-LBL. Subsequent bone marrow examination revealed positivity for the BCR::ABL1 (P210) fusion gene. Flow cytometry (FCM) and immunohistochemistry (IHC) confirmed that the tumor cells co-expressed T-lymphoid and myeloid antigens, and fluorescence *in situ* hybridization (FISH) detected BCR::ABL1 fusion signals in lymph node tissue, leading to the final diagnosis of *de novo* blast phase CML (BP-CML) with mixed phenotype (T-lineage and myeloid expression). The chromosomal karyotype evolved from the initial complex karyotype t(22;22) to t(9;22;22), revealing that the Philadelphia (Ph) chromosome translocation was the initiating event of the disease. The patient achieved molecular remission following chemotherapy with the Hyper-CVAD/MA regimen combined with a tyrosine kinase inhibitor (TKI). This case underscores the importance of differentiating BP-CML among adult Ph-positive T-lymphoblastic neoplasms and highlights the value of multi-platform integrated diagnosis for identifying atypical presentations.

## Case presentation

**Figure 1:**
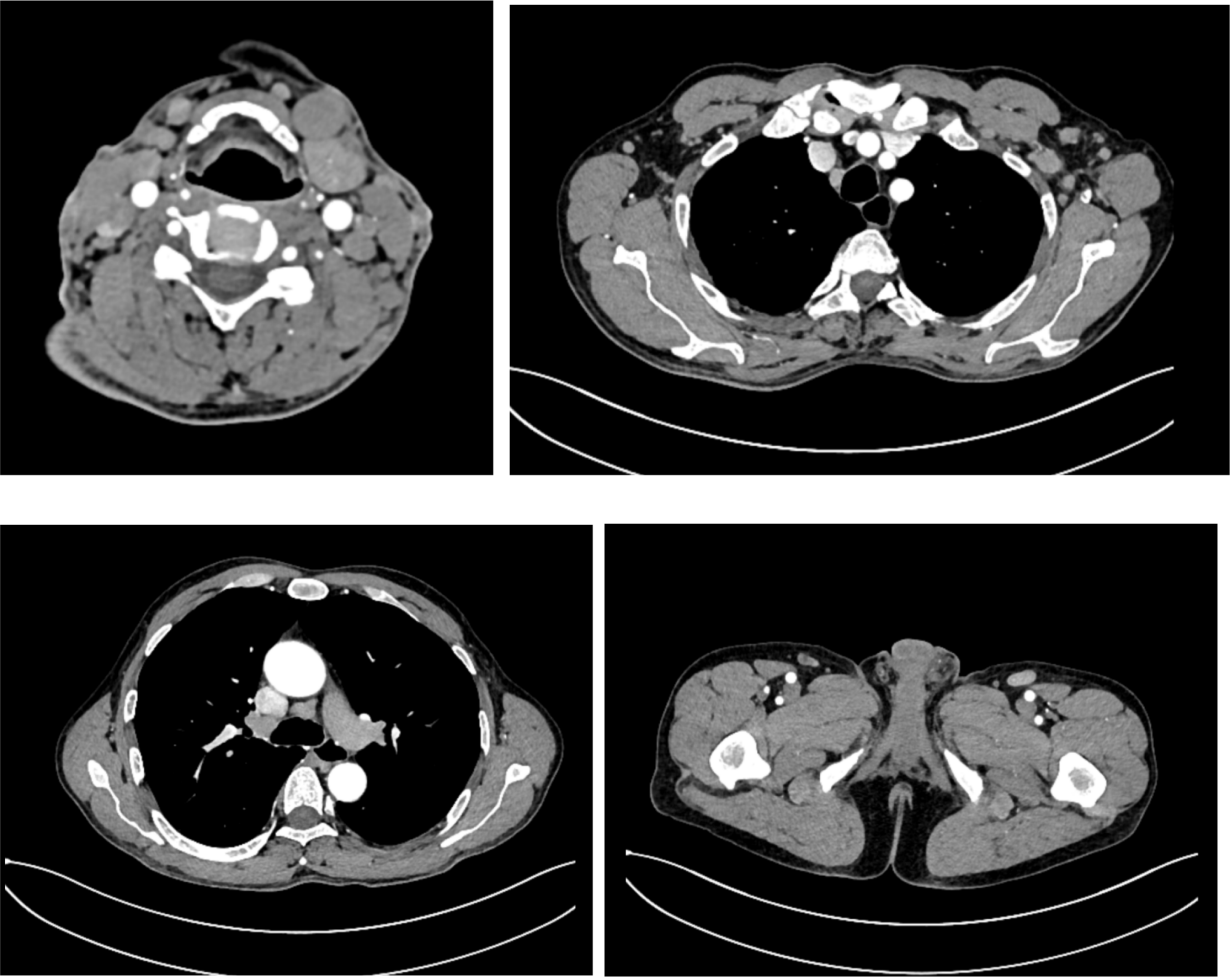
CT scan (January 16, 2024) showing multiple enlarged cervical lymph nodes, some forming confluent masses, along with enlarged nodes in the bilateral axillae, supraclavicular and infraclavicular regions, mediastinum, retroperitoneum, and bilateral inguinal regions.

**Figure 2:**
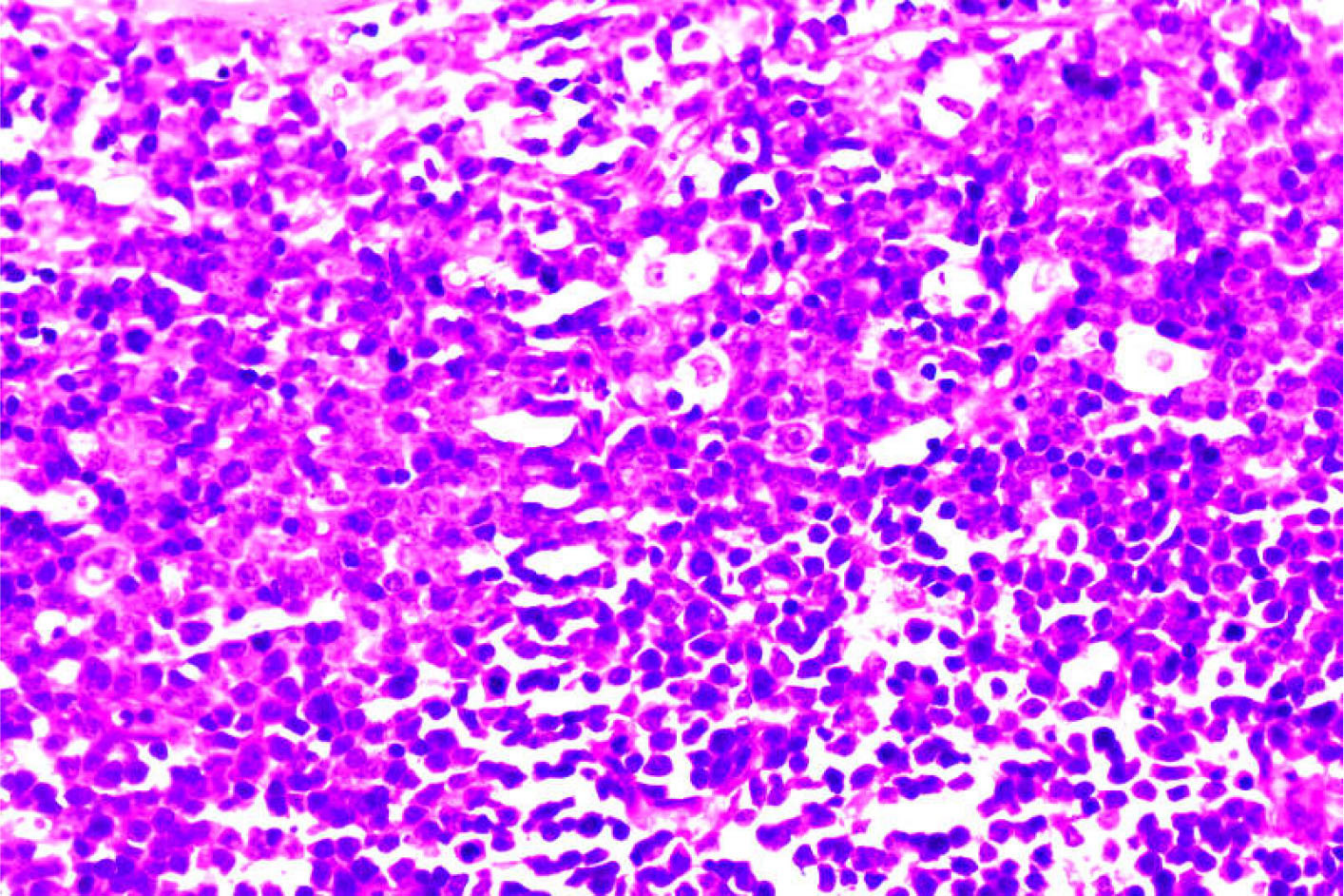
Cervical lymph node biopsy showing effacement of normal architecture by lymphoblastic infiltration (H&E, ×200). The morphologic and initial immunophenotypic features are consistent with T-LBL.

A 56-year-old male was admitted on January 15, 2024, after noticing left cervical masses for two weeks. Physical examination revealed multiple enlarged lymph nodes in the neck and submandibular region; they were firm and moderately mobile. The spleen was not palpable. Laboratory tests showed leukocytosis (white blood cell count 18.83 × 10^9^/L) with 72% neutrophils and 13% lymphocytes; markedly elevated lactate dehydrogenase (735 U/L), and slightly increased D-dimer (1.28 μg/mL). Computed tomography (CT) revealed generalized lymphadenopathy (**Figure 1**). Cervical lymph node biopsy showed effacement of normal nodal architecture. IHC demonstrated that tumor cells were positive for CD2, CD3, CD5, CD7, CD43, TdT, CD10, BCL-2, CD38, and CD30 (50%), with a Ki-67 proliferation index of 80%. They were negative for CD1a, CD138, κ, λ, CD56, CXCL13, CD4, PD1, CD8, CD20, Pax-5, Bcl-6, CKp, CyclinD1, ALK, TIA1, MUM1, c-Myc, S100, and CD34. CD79a showed focal weak positivity. CD21 and CD23 highlighted residual scattered follicular dendritic cell networks. EBER *in situ* hybridization was negative. These findings led to the preliminary diagnosis of T-LBL (**Figure 2**). Given progressive lymphadenopathy, the patient was started on Hyper-CVAD chemotherapy.

**Figure 3:**
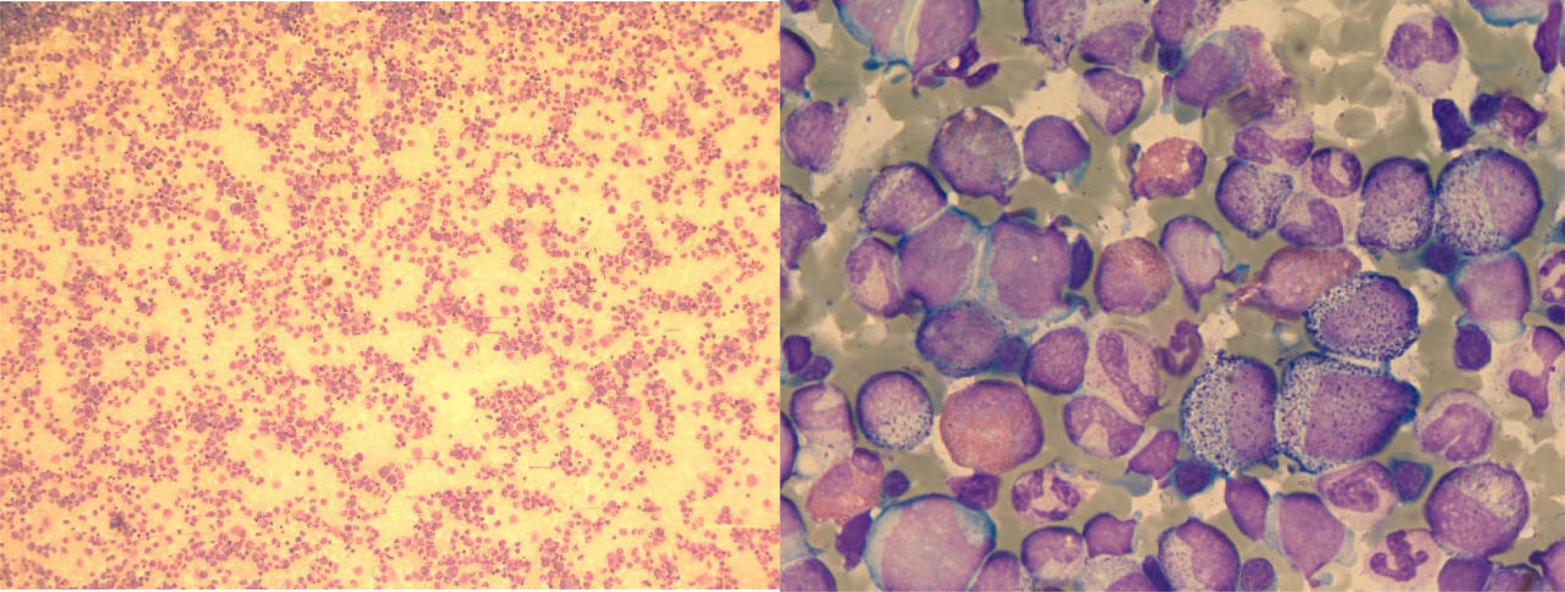
Bone marrow cytomorphology (January 29, 2024) showing extreme hyperplasia, predominance of myelocytes and metamyelocytes, increased eosinophils, elevated blasts (11.5%), and mature lymphocytes.

**Figure 4:**
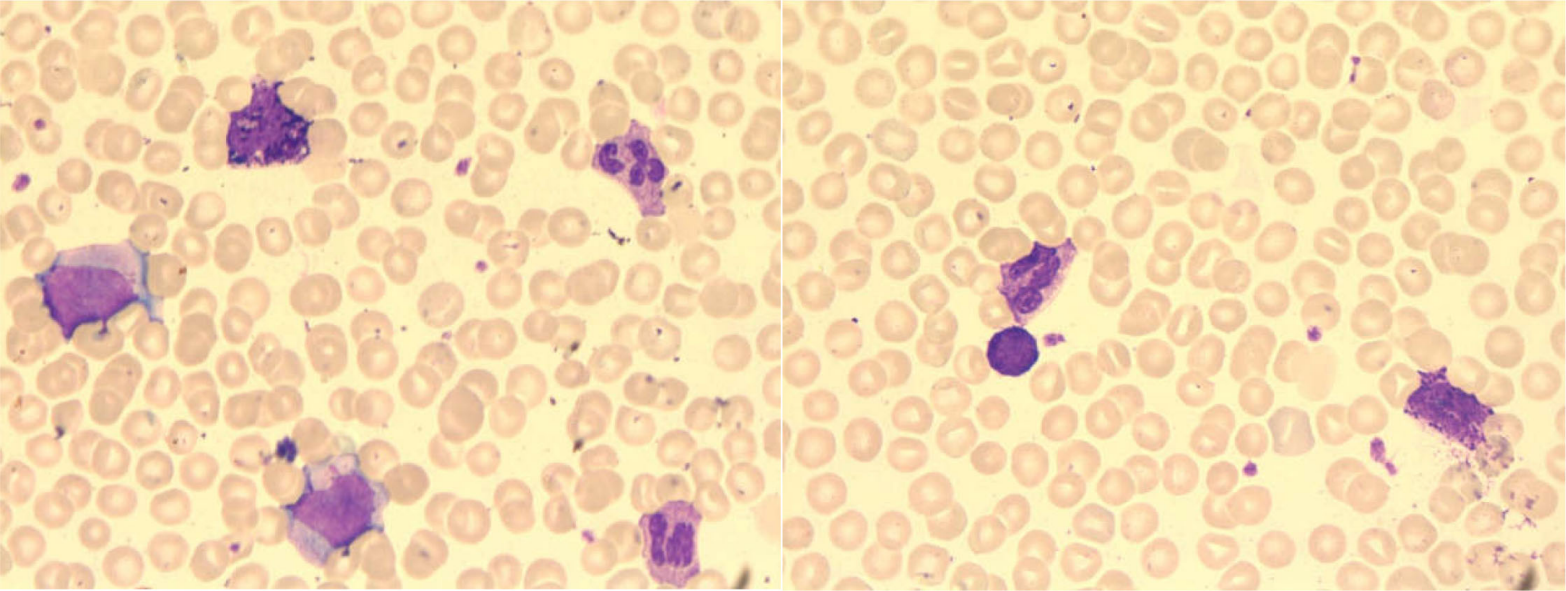
Peripheral blood smear (January 29, 2024) showing leukocytosis, elevated myeloid lineage ratio, increased myeloid cells, immature granulocytes at various stages, and 3% basophils.

**Figure 5:**
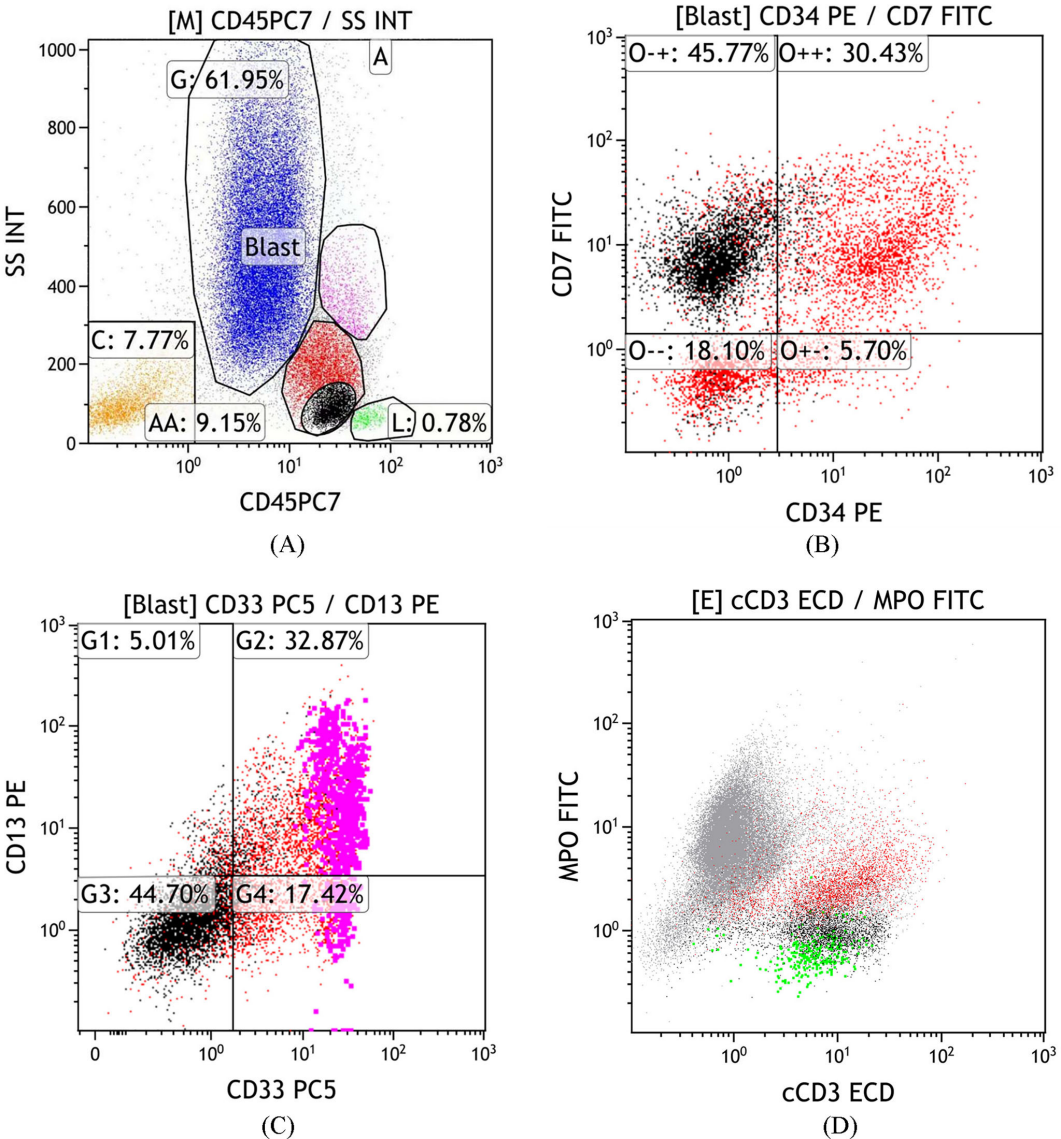
Bone marrow FCM (January 29, 2024). Two distinct blast populations were identified: pure T-lymphoid blasts (9.2%, black) and mixed phenotype (myeloid/T-lymphoid) blasts (7.6%, red). **(A)** CD45/SSC plot showing clear separation of the two populations by gating on CD45 and SSC; **(B)** CD34 vs. CD7 expression; **(C)** CD2 vs. CD117 expression; **(D)** MPO vs. cCD3 expression, illustrating the distinct immunophenotypic profiles of the two blast populations.

**Figure 6:**
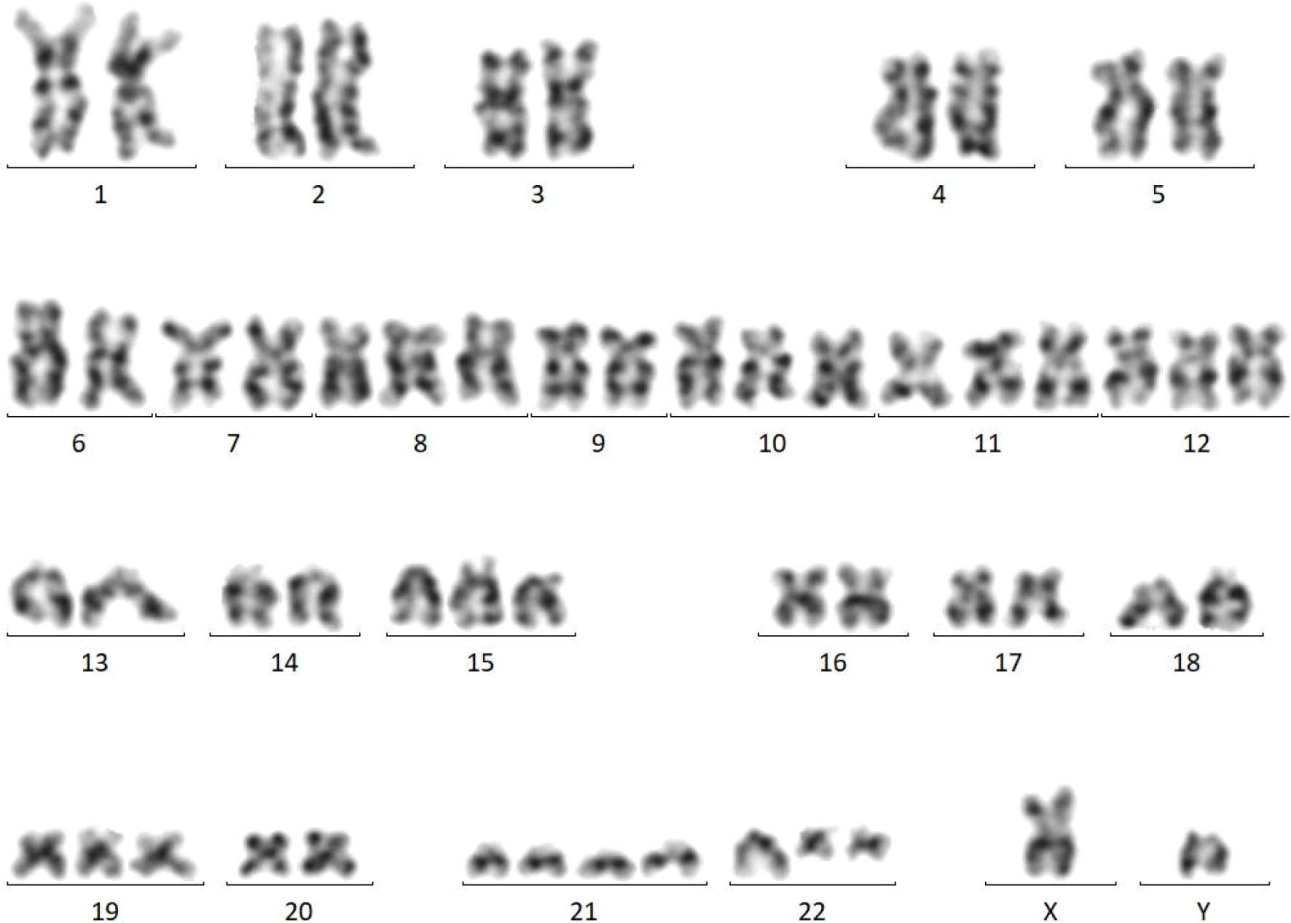
Bone marrow karyotype (January 29, 2024) showing 46,XY,t(22;22) (q11.2;q13) (17)/54,idem,+8,+10,+11,+12,+19,+21,+der(22)t(22;22) (8).

Subsequent test results revealed abnormalities. Bone marrow cytomorphology showed hypercellularity, normal granulocytic series with predominant myelocytes and metamyelocytes, increased eosinophils, elevated blasts (11.5%), and mature lymphocytes (**Figure 3**). Peripheral blood smear demonstrated marked leukocytosis (white blood cell count 17.5 × 10^9^/L), the granulocytic series was elevated with immature granulocytes at all stages, and basophils accounted for 3% (**Figure 4**). Bone marrow FCM detected a 16.8% blast population, comprising two distinct subsets: 9.2% pure T-lymphoid blasts (CD7+CD34-HLA-DR-CD10-CD20-CD19-CD14-CD13-CD33-CD2+CD117-CD15-CD11B-CD64-CD56+CD38+CD4-CD8-CD3-CD45 ± MPO-cCD3+) and 7.6% mixed phenotype (myeloid/T-lymphoid) blasts (CD7+CD34+HLA-DR-CD10-CD20-CD19-CD14-CD13 ± CD33 ± CD2+CD117+CD15-CD11B-CD64-CD56+CD38+CD4-CD8-CD3-CD45 ± MPO+cCD3+ with slightly increased SSC) (**Figure 5**). Crucially, the BCR::ABL1 (P210) fusion gene was detected with a quantitative value of 92.87%, a finding inconsistent with typical T-LBL. Chromosomal karyotype analysis at initial diagnosis showed 46,XY,t(22;22)(q11.2;q13) (17)/54,idem,+8,+10,+11,+12,+19,+21,+der(22)t(22;22) (8) (**Figure 6**), rather than the classic t(9;22). The patient was discharged after completing the first cycle of Hyper-CVAD.

**Figure 7:**
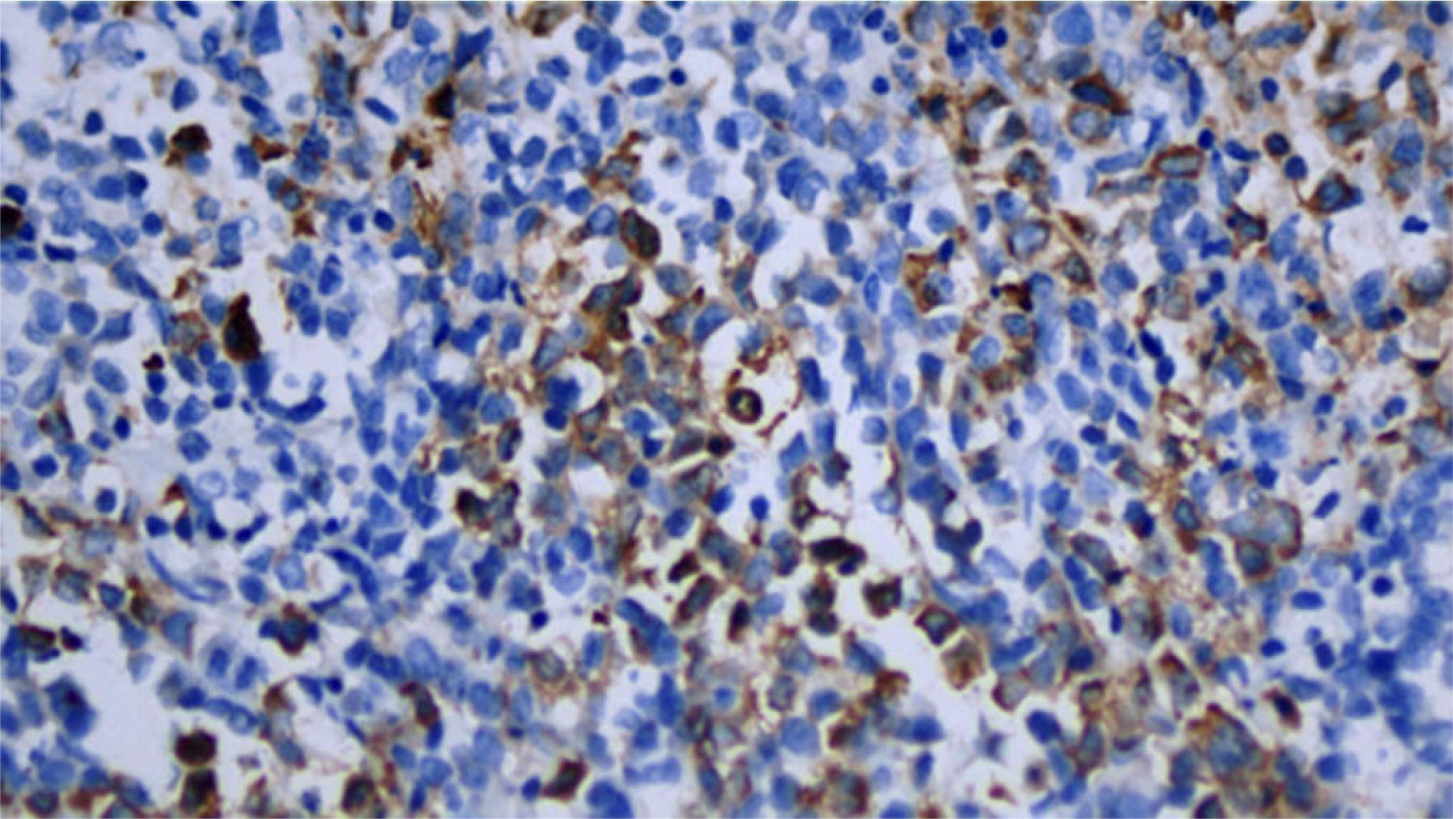
MPO immunohistochemistry of the lymph node biopsy specimen (February 29, 2024) showing approximately 25% of tumor cells positive for MPO (original magnification ×200).

**Figure 8:**
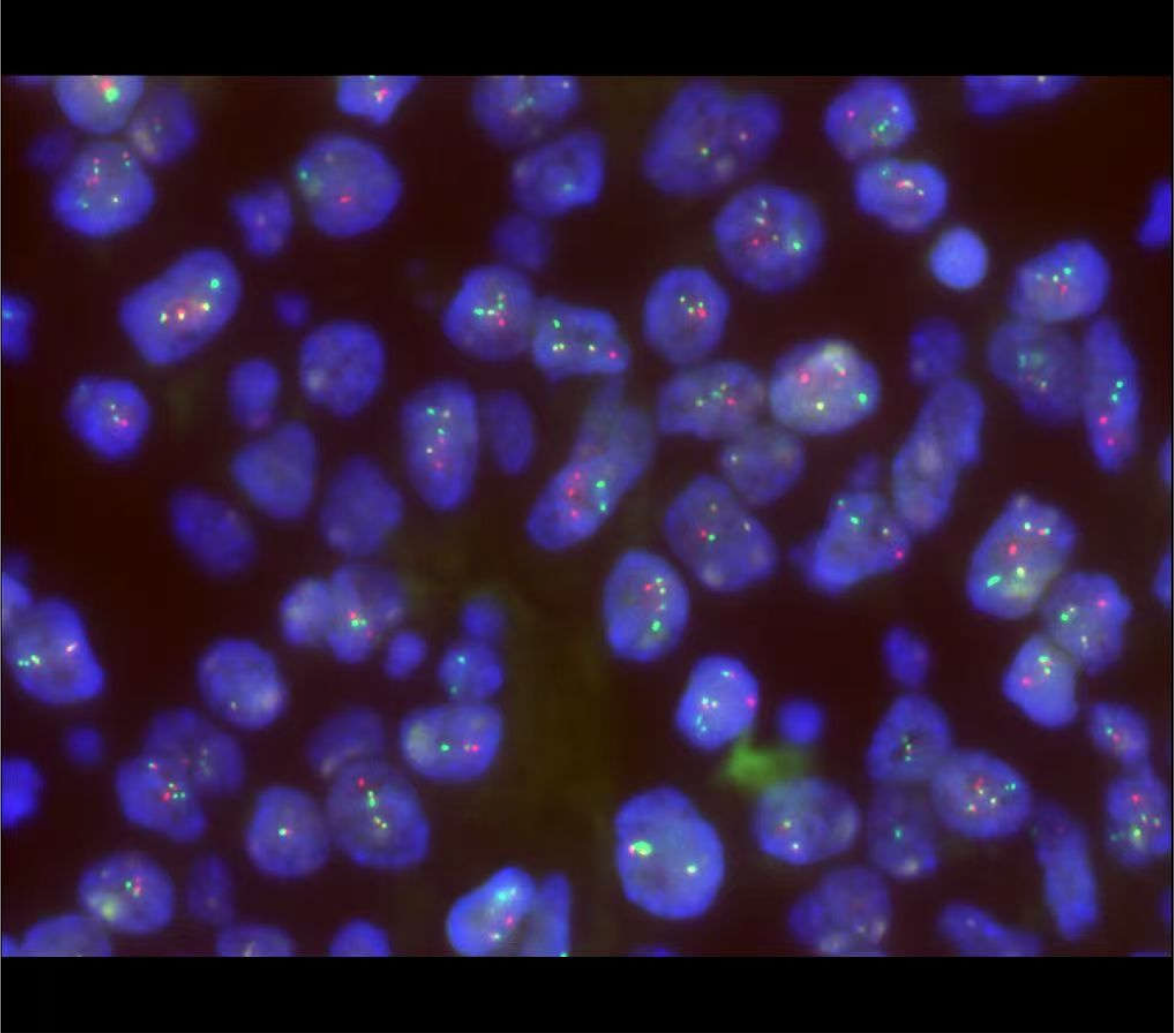
FISH analysis of the paraffin-embedded lymph node biopsy specimen (February 29, 2024) using dual-color dual-fusion BCR::ABL1 probes. Positive fusion signals (yellow) are visible in tumor cell nuclei, indicating BCR::ABL1 rearrangement.

**Figure 9:**
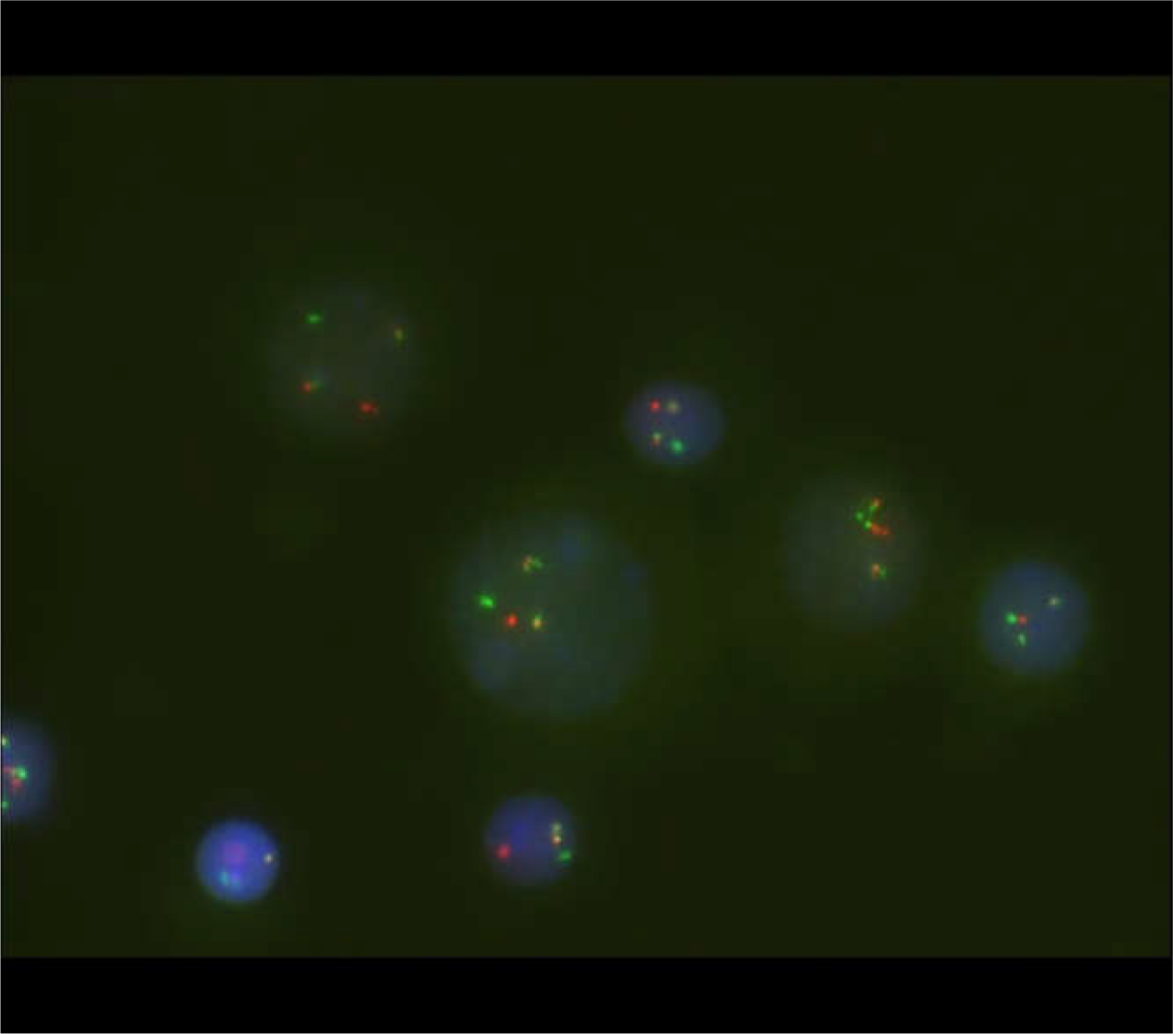
FISH analysis of bone marrow cells (February 29, 2024) using dual-color dual-fusion BCR::ABL1 probes. Positive fusion signals (yellow) are present in interphase nuclei, confirming BCR::ABL1 rearrangement.

The patient was readmitted on February 29, 2024, for the next treatment phase. To further clarify the diagnosis, additional studies were performed. On the lymph node biopsy specimens, myeloperoxidase(MPO) immunohistochemical staining showed that approximately 25% of tumor cells expressed myeloid markers (**Figure 7**), further supporting a mixed phenotype. To determine whether the BCR::ABL1 fusion was present despite the atypical t(22;22) karyotype and whether the lymph node involvement and bone marrow findings originated from the same clonal process, we performed FISH using dual-color dual-fusion BCR::ABL1 probes on both lymph node and bone marrow specimens. This demonstrated definitive fusion signals in the tumor cell area of the lymph node (**Figure 8**) and positivity in 93% of bone marrow cells (**Figure 9**), confirming the presence of the Ph chromosome in both compartments and supporting a clonal relationship between the lymphomatous presentation and the myeloid process. Bone marrow studies were repeated during this admission. Cytomorphology showed no significant abnormalities. Minimal residual disease (MRD) assessment by FCM detected 1.6% abnormal cells with a T-lymphoid phenotype (CD2+CD5+CD7+CD3-CD19-CD56/CD16+CD99+CD38+CD45±). Of note, at this time the diagnosis was still evolving, and based on the initial impression of T-LBL, the MRD panel had been designed based on the initial impression of T-LBL and therefore did not include myeloid markers. Thus, while this result confirmed residual T-lymphoid blasts, it could not assess whether mixed phenotype or myeloid blasts also persisted. During this admission, the patient received MA chemotherapy (methotrexate + cytarabine).Oral TKI (flumatinib 600mg once daily) was initiated after recovery from myelosuppression.

**Figure 10:**
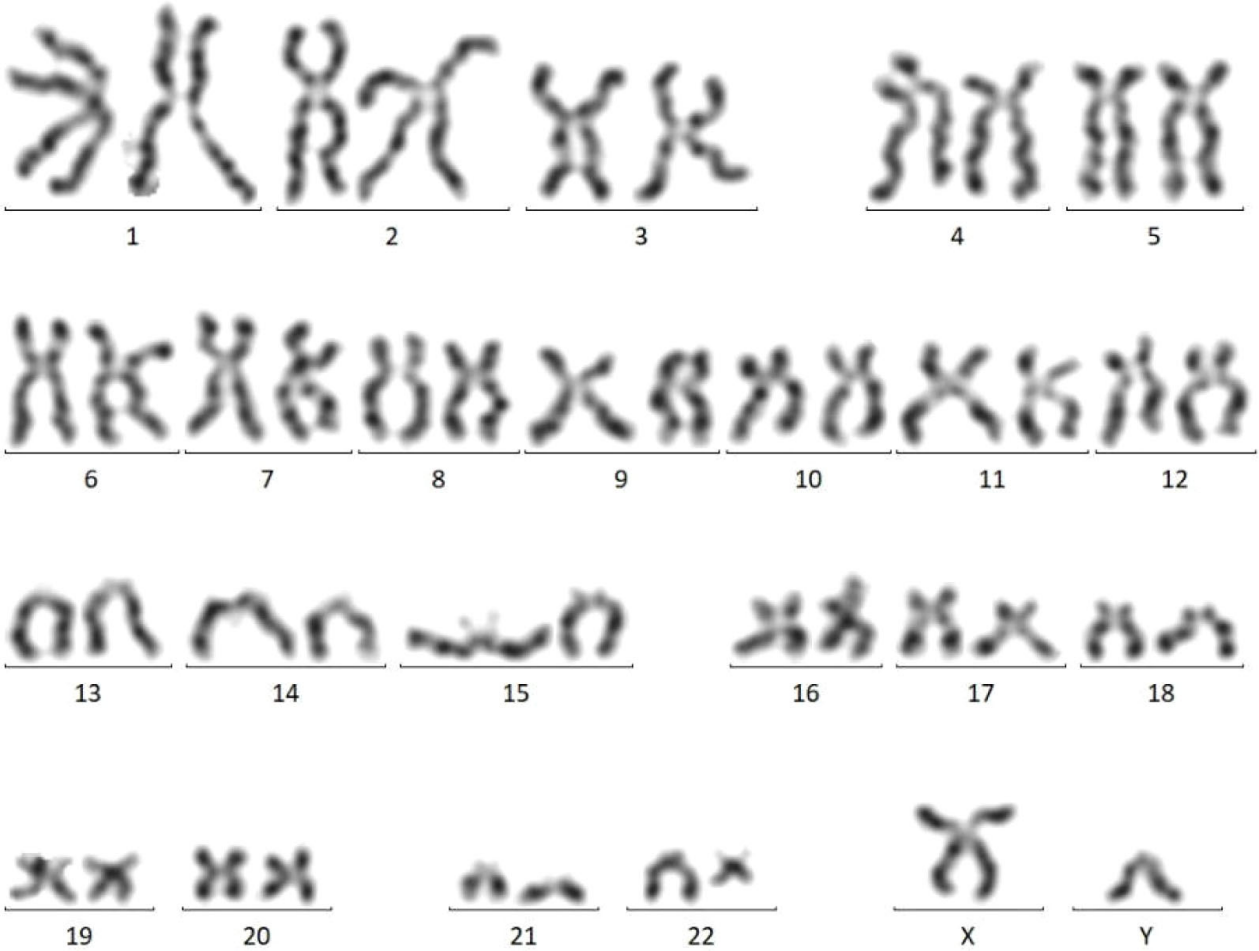
Bone marrow karyotype (May 17, 2024) showing 46,XY,t(9;22;22) (q34.1;q11.2;q13) (1)/46,XY (29).

The patient was readmitted for re-evaluation on May 17, 2024. At this time, bone marrow chromosomal karyotype had evolved to 46,XY,t(9;22;22)(q34.1;q11.2;q13) (1)/46,XY (29) (**Figure 10**). This finding suggests that the classic t(9;22) was likely the initiating event, with the t(22;22) representing a complex rearrangement that occurred subsequently, on this genetic background.

The differential diagnosis for this case primarily involves three possibilities: Ph-positive T-LBL, mixed-phenotype acute leukaemia(MPAL) with BCR::ABL1 fusion, and BP-CML.

Although the lymph node biopsy demonstrated effaced architecture with tumor cells expressing CD3, CD5, CD7, and TdT, consistent with T-LBL, primary Ph-positive T-LBL itself is exceedingly rare (1).

Furthermore, IHC for MPO on the lymph node specimen revealed approximately 25% of tumor cells to be MPO-positive. The presence of this lineage-specific myeloid marker makes a diagnosis of primary T-LBL difficult to sustain (2, 3).FCM detected a population comprising 7.6% of nucleated cells co-expressing cytoplasmic CD3 and MPO. This immunophenotype does not exclude the possibility of T/myeloid MPAL; however, the diagnosis of MPAL requires≥20% blasts in the bone marrow or peripheral blood (4, 5). In this patient, the blast count was 16.8%. Additionally, peripheral blood showed leukocytosis with circulating myeloid precursors at various stages, while the bone marrow was hypercellular with increased eosinophils. These findings are more suggestive of a background of CML rather than typical *de novo* MPAL. Critically, the diagnosis of MPAL with BCR::ABL1 fusion requires exclusion of BP-CML (5).

According to World Health Organization criteria, the presence of extramedullary blast proliferation is sufficient for a diagnosis of BP-CML (6). The lymph node biopsy from this patient showed tumor cells with lymphoblastic morphology, meeting this criterion for blast phase. Further evidence supporting BP-CML includes the presence of a BCR-ABL fusion gene, a complex karyotype at diagnosis, the emergence of a t(9;22;22) translocation following treatment demonstrating clonal evolution. These features are consistent with the biological behavior of CML. This patient presented in blast phase with no evidence of a preceding chronic phase, consistent with *de novo* BP-CML (7, 8).

In conclusion, based on the integrated clinical and laboratory evidence, a diagnosis of *de novo* BP-CML with mixed phenotype (T-lineage and myeloid expression) is favored.

**Figure 11:**
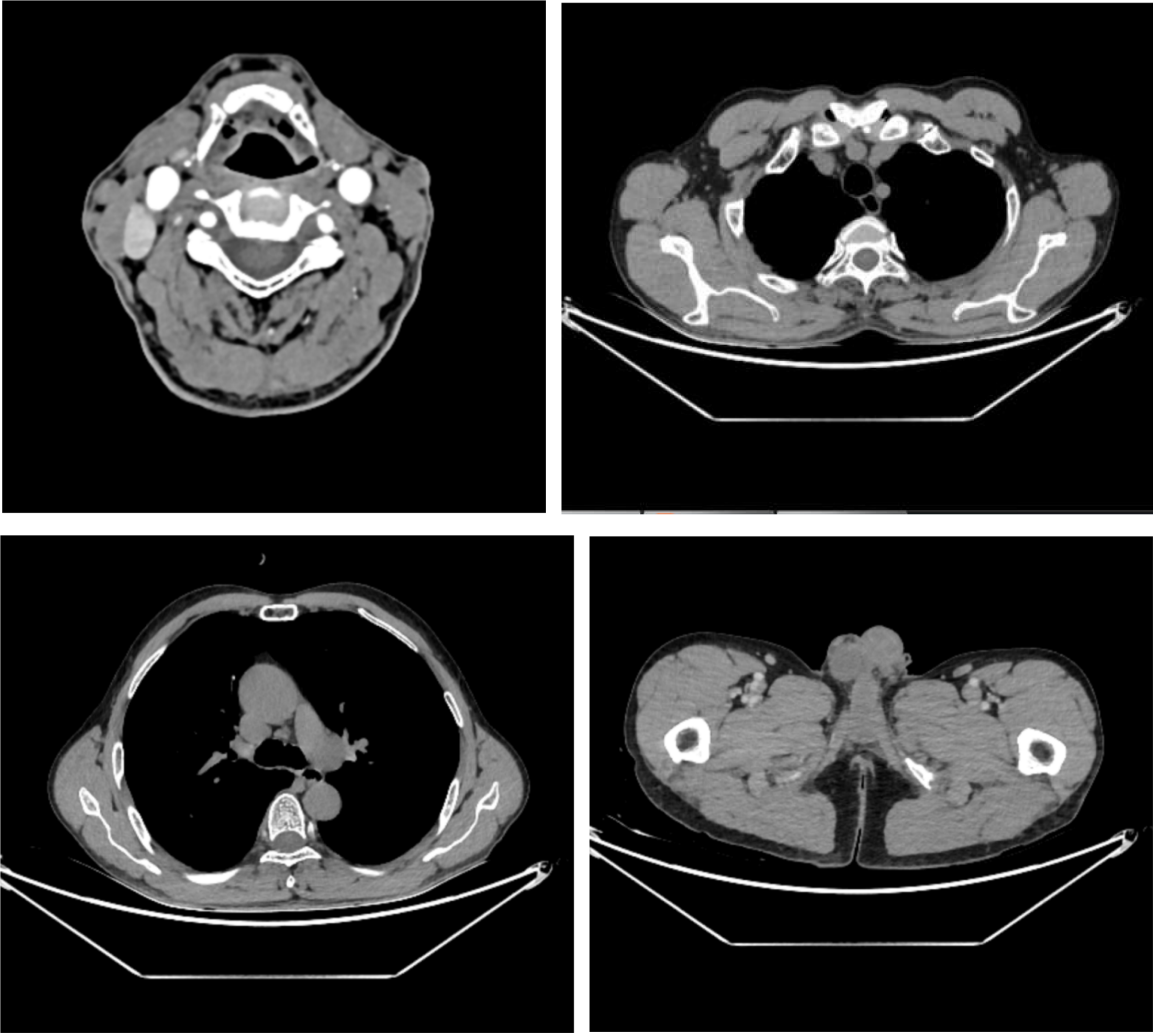
CT scan (July 15, 2025) showing normal-sized lymph nodes in the bilateral cervical, axillary, supra- and infraclavicular fossae, mediastinum, abdominal cavity, retroperitoneum, and bilateral inguinal regions.

The patient received one course of Hyper-CVAD/MA chemotherapy followed by TKI therapy, resulting in resolution of lymphadenopathy and normalization of blood counts, further supporting the diagnosis. The patient continues on TKI treatment. A follow-up CT scan in July 2025 showed no significant lymphadenopathy (**Figure 11**). Bone marrow FCM using a comprehensive antibody panel covering myeloid, T-lymphoid, and B-lymphoid lineages showed no abnormal cells. The karyotype was normal, and BCR::ABL1 (P210) was 0.54%. Re-evaluation in October 2025 showed BCR::ABL1 (P210) at 0.1037%, and no ABL kinase mutations were detected during treatment (**Table 1**). The patient remains clinically well and continues daily activities without limitations, but has consistently declined allogeneic hematopoietic stem cell transplantation(allo-HSCT) for personal reasons.

**Table 1:** Timeline of key laboratory findings and treatment evolution during the case management.

Time point	Immunophenotype (FCM/IHC)	Cytogenetics/FISH	Molecular findings	Therapy
January 15, 2024 (Initial Diagnosis)	Lymph node IHC: CD3(+), CD5(+), CD7(+), TdT(+) (consistent with T-LBL phenotype); Bone marrow FCM: 7.6% myeloid/T mixed phenotype blasts and 9.2% T-lineage phenotype blasts	Bone marrow karyotype: 46,XY,t(22;22)(q11.2;q13) (17)/54,idem,+8,+10,+11,+12,+19,+21, +der(22)t(22;22) (8)	BCR::ABL1 (P210) quantitative: 92.87%	Preliminary diagnosis of T-LBL; initiation of Hyper-CVAD regimen chemotherapy
February 29, 2024 (Final Diagnosis)	Lymph node IHC: Approximately 25% of tumor cells MPO(+); Bone marrow FCM MRD: 1.6% abnormal cells	Lymph node FISH: BCR::ABL1 fusion signals positive; Bone marrow FISH: BCR::ABL1 positive (93%)	–	MA regimen chemotherapy followed by TKI (flumatinib 600mg once daily)
May 17, 2024 Examination during treatment)	Bone marrow FCM MRD: <10^-4^	Bone marrow karyotype: 46,XY,t(9;22;22)(q34.1;q11.2;q13) (1)/46,XY (29)	BCR::ABL1 (P210) quantitative: 53.41%	Continued TKI
July 15, 2025 (Response evaluation)	Bone marrow FCM: No phenotypically abnormal cells	Bone marrow karyotype: 46,XY (20)	BCR::ABL1 (P210) quantitative: 0.54%; No ABL kinase mutations detected	Continued TKI
October 28, 2025(Response evaluation)	–	–	BCR::ABL1 (P210) quantitative: 0.1037%; No ABL kinase mutations detected	Continued TKI

## Discussion

This case describes a rare presentation of BP-CML and offers insights into its disease biology.

Cytogenetically, this case exhibited a dynamic evolution from t(22;22) to t(9;22;22). Complex karyotype evolution is a characteristic feature of CML in blast phase (9). The initial complex karyotype, featuring hyperdiploidy and additional abnormalities, reflects the genomic instability typical of advanced disease (7). The absence of the classic t(9;22) at initial diagnosis may be explained as follows: a conventional t(9;22) translocation likely occurred first, forming a classic Ph chromosome that may not have been cytogenetically detectable or may have undergone subsequent recombination. This derivative chromosome 22, already harboring the ABL1 gene, may then have participated in a secondary translocation t(22;22) with another normal chromosome 22, with breakpoints at 22q11.2 and 22q13. This complex secondary translocation could have generated a new derivative chromosome, der(22)t(22;22), which appears to retain the BCR::ABL1 fusion gene—supported by the presence of BCR::ABL1 fusion signals in tumor cells detected by FISH in both bone marrow and lymph node tissues. Thus, despite the apparent atypical translocation partner (not chromosome 9) in the final karyotype, the molecular consequence is presumably identical to that of the classic Ph chromosome, resulting in the expression of the BCR::ABL1 fusion protein with sustained tyrosine kinase activity. The emergence of the three-way translocation t(9;22;22) (q34.1;q11.2;q13) following treatment provides insight into possible clonal evolution. This pattern aligns with the clonal evolution theory, whereby therapeutic pressure may eliminate genomically unstable clones, potentially allowing the emergence of subclones closer to the disease origin (10). Studies have suggested that the classic t(9;22) is the initiating genetic event in CML, and additional abnormalities may serve as markers of disease progression (11).

Only about 5% of patients with CML present in accelerated or blast phase at diagnosis (12). In this case, lymphadenopathy was the initial manifestation of CML, and the tumor cells exhibited a mixed T-cell and myeloid phenotype, which is extremely rare clinically (12, 13). *In situ* confirmation of BCR::ABL1 within lymph node tumor cells by FISH provides experimental evidence for the differentiation potential of CML pluripotent stem cells. It is thought that BCR::ABL1−positive clones can differentiate into multiple hematopoietic lineages, and CML stem cells retain both myeloid and lymphoid differentiation potential (14). Lineage infidelity represented another notable feature. FCM demonstrated a myeloid/T-lymphoid mixed phenotype, and MPO immunohistochemistry revealed that a subset of tumor cells co-expressed myeloid markers. These findings suggest that the neoplastic cells retained stem cell properties despite exhibiting T-lineage differentiation. Studies have shown that the BCR::ABL1 signaling pathway may induce differentiation arrest by affecting key transcription factors and can disrupt normal hematopoietic differentiation through epigenetic mechanisms (15, 16).

Mixed phenotype BP-CML is clinically rare and lacks standardized treatment guidelines. Based on general principles for managing BP-CML and published reports (7, 17, 18), treatment should involve selecting an appropriate acute leukemia induction chemotherapy regimen according to the dominant blast lineage (e.g., the Hyper-CVAD regimen for lymphoid lineage) combined with second- or third-generation TKIs to continuously inhibit the BCR::ABL1 driver. The primary goal is to achieve remission and revert to chronic phase, ideally allowing eligible patients to proceed to allo-HSCT, which remains the only potentially curative option. This was recommended to the patient but declined for personal reasons.

The patient’s sustained molecular response to chemotherapy and TKI therapy validates the diagnosis. The concordant cytogenetic and molecular remission supports the treatment strategy’s efficacy and confirms that, despite its lymphoma-like presentation, the disease was fundamentally driven by BCR::ABL1.

This case documents the rare presentation of BP-CML mimicking T-LBL, accompanied by complex karyotypic evolution. It underscores the need to include BP-CML in the differential diagnosis of adult patients with Ph-positive T-lymphoblastic neoplasms. Lineage infidelity and complex karyotypes may signal advanced disease. The integrated application of multi-platform diagnostic technologies is essential for the accurate identification of such cases. Further studies are warranted to investigate optimal treatment strategies and prognostic factors for this patient population.

## Data Availability

The original contributions presented in the study are included in the article/supplementary material. Further inquiries can be directed to the corresponding authors.
